# LGI1 acts presynaptically to regulate excitatory synaptic transmission during early postnatal development

**DOI:** 10.1038/srep21769

**Published:** 2016-02-16

**Authors:** Morgane Boillot, Chun-Yao Lee, Camille Allene, Eric Leguern, Stéphanie Baulac, Nathalie Rouach

**Affiliations:** 1 Sorbonne Universités, UPMC Univ Paris 06, UM 75, ICM, F-75013 Paris, France; 2 INSERM, U1127, ICM, Paris, F-75013 Paris, France; 3 CNRS, UMR 7225, ICM, Paris, F-75013 Paris, France; 4 ICM, Paris, F-75013 Paris, France; 5 Neuroglial Interactions in Cerebral Physiopathology, Center for Interdisciplinary Research in Biology, Collège de France, CNRS UMR 7241, INSERM U1050, Labex Memolife, PSL Research University, F-75005 Paris, France; 6 Département de Génétique et de Cytogénétique, AP-HP Groupe hospitalier Pitié-Salpêtrière, F-75013 Paris, France

## Abstract

The secreted leucine-rich glioma inactivated 1 (LGI1) protein is an important actor for human seizures of both genetic and autoimmune etiology: mutations in *LGI1* cause inherited temporal lobe epilepsy, while LGI1 is involved in antibody-mediated encephalitis. Remarkably, *Lgi1-*deficient (*Lgi1*^−/−^) mice recapitulate the epileptic disorder and display early-onset spontaneous seizures. To understand how Lgi1-deficiency leads to seizures during postnatal development, we here investigated the early functional and structural defects occurring before seizure onset in *Lgi1*^−/−^ mice. We found an increased excitatory synaptic transmission in hippocampal slices from *Lgi1*^−/−^ mice. No structural alteration in the morphology of pyramidal cell dendrites and synapses was observed at this stage, indicating that Lgi1-deficiency is unlikely to trigger early developmental abnormalities. Consistent with the presynaptic subcellular localization of the protein, Lgi1-deficiency caused presynaptic defects, with no alteration in postsynaptic AMPA receptor activity in *Lgi1*^−/−^ pyramidal cells before seizure onset. Presynaptic dysfunction led to increased synaptic glutamate levels, which were associated with hyperexcitable neuronal networks. Altogether, these data show that Lgi1 acts presynaptically as a negative modulator of excitatory synaptic transmission during early postnatal development. We therefore here reveal that increased presynaptic glutamate release is a key early event resulting from Lgi1-deficiency, which likely contributes to epileptogenesis.

Mutations in the *LGI1* (Leucine-rich, glioma inactivated 1) gene account for nearly half of families with autosomal dominant epilepsy with auditory features (ADEAF)^1^,^2^. ADEAF refers to an inherited epileptic condition of adolescent/adulthood-onset clinically characterized by lateral temporal lobe seizures with auditory features^3^. Besides, LGI1 autoantibodies are found in the sera or cerebrospinal fluids (CSF) in a fraction of patients with limbic encephalitis, an adult-onset neuropsychiatric disorder associating cognitive impairment and seizures^4^,^5^.

While the role of Lgi1 in epilepsy remains to be further defined, three main hypotheses underlying Lgi1 function have emerged: 1/Lgi1 may potentiate excitatory synaptic transmission through modulation of postsynaptic AMPA receptors (AMPARs) and binding to Adam22 and 23 (A Disintegrin And Metalloprotease domain) transmembrane proteins^6^; 2/Lgi1 may inhibit inactivation of the presynaptic voltage-gated potassium channel subunit Kv1.1^7^; 3/Lgi1 may play a role in the maturation of glutamatergic neurons, as shown in mice overexpressing a truncated form of Lgi1 and presenting immature pruning of spines and dendrites^8^,^9^. Moreover, in *vitro*, Lgi1 was shown to enhance neuronal growth^10^.

Mutations of *LGI1* are likely to lead to a loss-of-function^11^. Our group and two others have generated Lgi1-deficient (*Lgi1*^−/−^) mice, that recapitulate some features of the epileptic disorder^12^,^13^,^14^. While no seizures are detected at postnatal day 9 (P9), *Lgi1*^−/−^ mice display frequent spontaneous epileptic seizures from P10, and die within the first three postnatal weeks^12^. Electrophysiological recordings from hippocampal slices of *Lgi1*^−/−^ mice suggested a role for Lgi1 in controlling transmission at excitatory synapses. However, because of contradictory results, it remains unclear whether Lgi1 promotes or reduces excitatory synaptic transmission, and whether it acts pre or postsynaptically^13^,^14^. Recently, our work on conditional *Lgi1*-deficient mice demonstrated that deletion of *Lgi1* restricted to pyramidal cells is sufficient to trigger spontaneous epileptic activities, thus confirming *in vivo* the major role of excitatory neurons in seizure emergence^15^.

Here, we aimed to further get insight into the pathogenic mechanism underlying the origin of seizures in LGI1-related epilepsies, independently of circuit damage due to seizure occurrence. We therefore searched for possible structural and functional defects in hippocampal slices from *Lgi1*^−/−^ mice at early postnatal stages (P8–P9), when no seizures are detected. At this stage, no morphological rearrangements at both synaptic and dendritic levels were evident in *Lgi1*^−/−^ mice. However synaptic excitatory transmission was enhanced, independently of any postsynaptic effect. Instead, the increased glutamatergic synaptic transmission resulted from a presynaptic defect, which induced enhanced synaptic glutamate levels, and was associated with hippocampal network hyperexcitability.

## Results

### Lgi1-deficiency enhances hippocampal glutamatergic synaptic transmission

**Figure 1:**
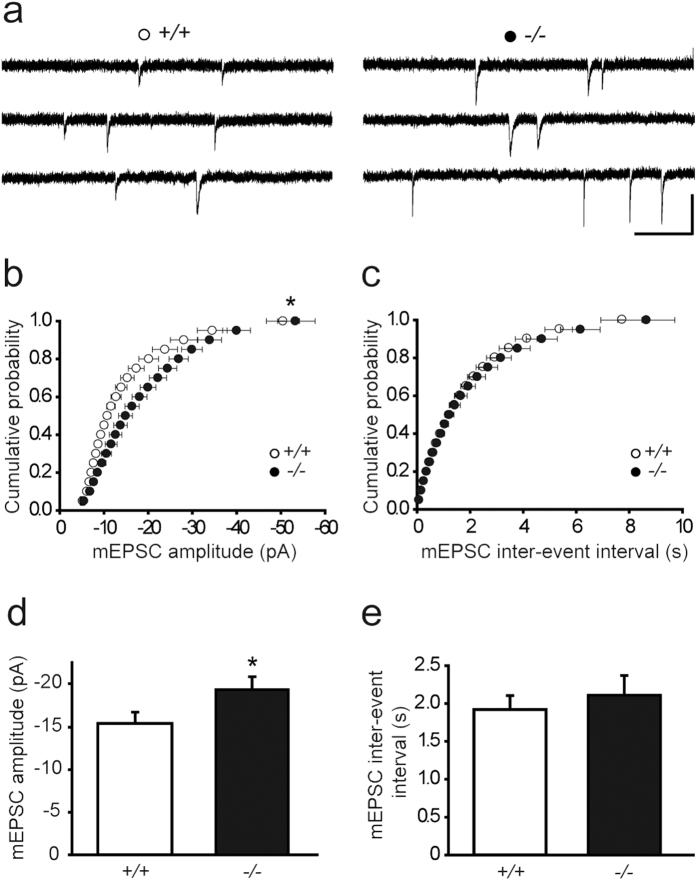
Hippocampal glutamatergic synaptic transmission is increased in *Lgi1*^−/−^ mice. Cumulative distributions and means of mEPSC amplitude (**b,d**) and inter-event interval (**c,e**), with sample traces above (**a**), in CA1 pyramidal cells from *Lgi1*^+/+^ (n = 18) and *Lgi1*^−/−^ mice (n = 20) at P8. Scale bar: 20 pA, 500 ms. mEPSC amplitude cumulative distribution and mean, but not inter-event interval, differ significantly between *Lgi1*^−/−^ and *Lgi1*^+/+^ mice (*p* < 0.05, Kolmogorov-Smirnov and *t*-test, respectively).

Deletion of *Lgi1* in mouse causes spontaneous seizures detected from P10^12^, and alters excitatory synaptic function during the active phase of epilepsy^13^,^14^. To get insight into the pathogenic mechanisms induced by Lgi1-deficiency, we investigated whether, before seizure onset, glutamatergic synaptic transmission is already affected in Lgi1-deficient mice. Analysis of AMPAR miniature excitatory postsynaptic currents (mEPSCs) from CA1 pyramidal cells revealed in P8 *Lgi1*^−/−^ mice a significant increase in amplitude of these events (*Lgi1*^−/−^, n = 20; *Lgi1*^+/+^, n = 18; *p* < 0.05, *t*-test; Fig. 1a,d). Remarkably, the cumulative amplitude probability showed an overall increase in mEPSC amplitudes in *Lgi1*^−/−^ pyramidal cells (*p* < 0.05, Kolmogorov-Smirnov; Fig. 1b), whereas no change in inter-event interval was detected (*Lgi1*^−/−^, n = 20; *Lgi1*^+/+^, n = 18; *p* > 0.05, Kolmogorov-Smirnov and *t*-test; Fig. 1c,e). Furthermore, mEPSCs also exhibited increased charge transfer (*Lgi1*^−/−^: 246.1 ± 22.2 pA*ms, n = 20; *Lgi1*^+/+^: 183.2 ± 14.2 pA*ms, n = 18; *p* < 0.05, *t*-test), with no change in rise time (*Lgi1*^−/−^: 2.36 ± 0.28 ms, n = 20; *Lgi1*^+/+^: 2.10 ± 0.18, n = 18; *p* > 0.05, *t*-test) and decay time (*Lgi1*^−/−^: 24.85 ± 2.10 ms, n = 20; *Lgi1*^+/+^: 22.48 ± 0.74, n = 18; *p* > 0.05, *t*-test), consistent with the increased amplitude of these events. Altogether these data show that mEPSCs have increased amplitude and transfer more charge per event with no change in kinetics in *Lgi1*^−/−^ mice, indicating that Lgi1-deficiency, at early postnatal stage, augments glutamatergic drive onto CA1 pyramidal cells.

### The dendritic and synaptic structure of pyramidal cells is not altered in *Lgi1*^−/−^ mice

**Figure 2:**
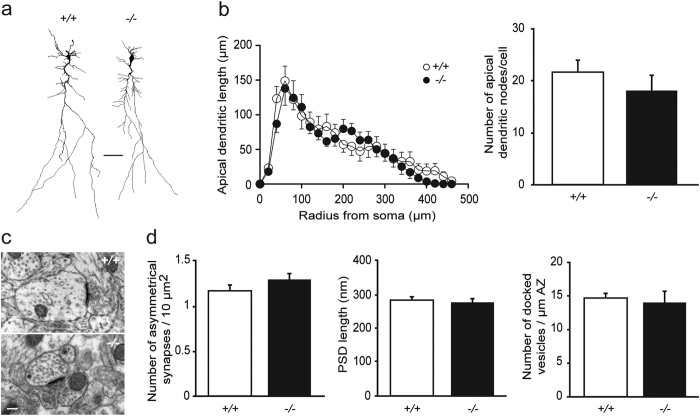
Dendritic arborization and excitatory synapses are unaltered in hippocampal pyramidal cells from *Lgi1*^−/−^ mice. (**a**) Representative dendritic morphology of neurobiotin-filled CA1 pyramidal cells from *Lgi1*^+/+^ and *Lgi1*^−/−^ mice at P9. Scale bar: 50 μm. (**b**) Sholl analysis reveals similar apical dendritic length (*p* > 0.05, two-way ANOVA) and number of apical dendritic nodes (*p* > 0.05, Mann-Whitney) between *Lgi1*^+/+^ (n = 9) and *Lgi1*^−/−^ mice (n = 14). (**c**) Electron micrographs of asymmetrical synapses in CA1 *stratum radiatum* from *Lgi1*^+/+^ and *Lgi1*^−/−^ mice at P8. Scale bar: 200 nm. (**d**) No change was found in asymmetrical synapse density (n = 2900 μm^2^ from 9 *Lgi1*^+/+^ and 6 *Lgi1*^−/−^ mice), PSD length (n = 40 synapses from 9 *Lgi1*^+/+^ and 6 *Lgi1*^−/−^ mice) and number of docked vesicles (n = 20 synapses from 9 *Lgi1*^+/+^ and 6 *Lgi1*^−/−^ mice) (*p* > 0.05, Mann-Whitney). PSD, postsynaptic density; AZ, active zone.

The number of dendrites and synapses of rodent CA1 pyramidal cells undergo substantial increase during the early postnatal period^16^. We therefore first hypothesized that the enhanced glutamatergic transmission occurring at early postnatal stage in *Lgi1*^−/−^ mice may reflect developmental alterations in neuronal modeling at dendritic and synaptic structures. Although no major neurodevelopmental abnormalities were reported in Lgi1-deficient mice^12^,^15^, subcellular morphological alterations have not yet been examined. We thus compared three-dimensional neuronal reconstructions of neurobiotin-filled CA1 pyramidal cells from *Lgi1*^−/−^ and *Lgi1*^+/+^ littermates at P9 (Fig. 2a). Sholl analysis of neuronal morphology (including soma size, dendritic volume and length, and number of dendritic nodes), revealed no difference between both genotypes. In particular, the length of apical dendrites and the number of apical nodes were unchanged in *Lgi1*^−/−^ mice compared to *Lgi1*^+/+^ mice (*Lgi1*^−/−^, n = 14; *Lgi1*^+/+^, n = 9; *p > 0.05*, respectively two-way ANOVA and Mann-Whitney; Fig. 2b), indicating that the arbor complexity is not altered in *Lgi1*^−/−^ mice at P9.

We next performed ultrastructural electron microscopy analysis of asymmetrical synapses in CA1 *stratum radiatum* at P8 (Fig. 2c). The density of asymmetrical synapses was comparable between *Lgi1*^−/−^ (n = 6) and *Lgi1*^+/+^ littermates (n = 9; *p* > 0.05, Mann-Whitney; Fig. 2d). Moreover, Lgi1-deficiency had no effect on both postsynaptic density length and number of presynaptic docked vesicles (*Lgi1*^−/−^, n = 6; *Lgi1*^+/+^, n = 9; *p* > 0.05, Mann-Whitney; Fig. 2d), indicating that functional maturation of excitatory synapses is normal at P8. Altogether, these data show that dendritic morphology and synaptic structure are normal in CA1 pyramidal cells from *Lgi1*^−/−^ mice aged P8-P9, suggesting that the enhanced excitatory transmission is not caused by early developmental defects in neuronal modeling.

### Lgi1-deficiency does not alter the density of postsynaptic AMPARs during early postnatal development

**Figure 3:**
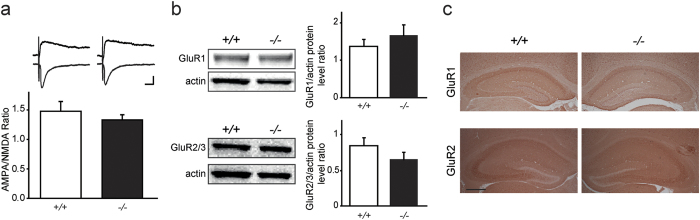
Postsynaptic AMPAR density is unchanged in *Lgi1*^−/−^ mice. (**a**) Similar ratios of AMPA to NMDA eEPSCs in CA1 pyramidal cells from P8 *Lgi1*^+/+^ (n = 6) and *Lgi1*^−/−^ mice (n = 7; *p* > 0.05, *t*-test), with sample traces above. Scale bars: 20 ms, 50 pA. (**b**) Top: Representative Western blot and quantification of GluR1 and actin bands showing similar GluR1 protein levels in the hippocampus of P8 *Lgi1*^+/+^ (n = 4) and *Lgi1*^−/−^ mice (n = 5; *p* > 0.05, *t*-test). Bottom: Representative Western blot and quantification of GluR2/3 and actin bands showing similar GluR2/3 protein levels in the hippocampus of P8 *Lgi1*^+/+^ (n = 3) and *Lgi1*^−/−^ mice (n = 3; *p* > 0.05, *t*-test). (**c**) Representative immunohistochemical staining for GluR1 (top) and extracellular GluR2 (bottom) subunits in hippocampal sections of *Lgi1*^+/+^ (n = 3) and *Lgi1*^−/−^ (n = 3) mice showing similar expression of the AMPAR subunits at P8. Scale bar: 400 μm.

The enhanced excitatory synaptic transmission in *Lgi1*^−/−^ mice may result from an increase in postsynaptic AMPAR density. We therefore tested whether the increased glutamatergic transmission was specific to AMPARs or similarly altered the AMPAR and NMDAR components of evoked EPSCs. The AMPA/NMDA ratio was unchanged in CA1 pyramidal cells from *Lgi1*^−/−^ mice (*Lgi1*^−/−^, n = 7; *Lgi1*^+/+^, n = 6; *p* > 0.05, *t*-test, Fig. 3a), indicating that the enhanced excitatory synaptic transmission equally affected AMPAR and NMDAR currents. This finding suggests that postsynaptic AMPAR density is unaltered in P8 *Lgi1*^−/−^ mice.

Consistent with our electrophysiological data, protein levels of the AMPAR GluR1 (*Lgi1*^−/−^, n = 5; *Lgi1*^+/+^, n = 4; *p* > 0.05, *t*-test) and GluR2/3 (*Lgi1*^−/−^, n = 3; *Lgi1*^+/+^, n = 3; *p* > 0.05, *t*-test) subunits were similar in the hippocampus of *Lgi1*^−/−^ and *Lgi1*^+/+^ littermates at P8 by Western blot (Fig. 3b). Likewise, GluR1 and GluR2 expression level revealed by immunostaining was not markedly changed in *Lgi1*^−/−^ compared to *Lgi1*^+/+^ hippocampal slices (Fig. 3c). Altogether, these data suggest that the enhanced synaptic transmission in *Lgi1*^−/−^ mice does not result from an increase in postsynaptic AMPAR density at early postnatal stage.

### *Lgi1*^−/−^ mice present presynaptic defects with increased synaptic glutamate levels

**Figure 4:**
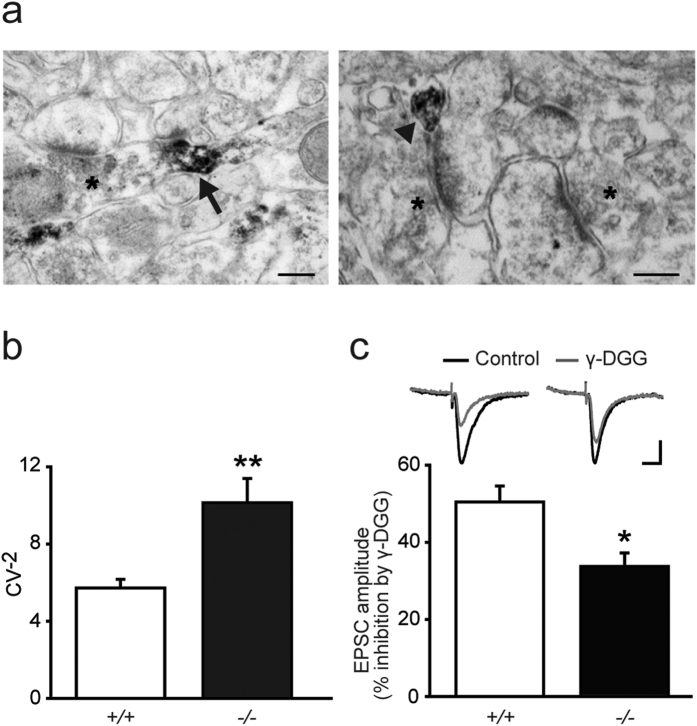
Presynaptic alterations lead to increased synaptic glutamate levels in *Lgi1*^−/−^ mice. (**a**) Hippocampal electron micrographs from an *Lgi1*^+/+^ mouse showing the subcellular localization of Lgi1, immunostained with patient CSF, in presynaptic elements (arrow) and thin neurites (arrowhead). Non-reactive synaptic contacts are labeled with asterisks. Scale bars: 200 nm. (**b**) Coefficient of variation (CV) analysis of AMPAR eEPSC amplitude reveals an increase in CV^−2^ in CA1 pyramidal cells from P8 *Lgi1*^−/−^ (n = 23) compared to *Lgi1*^+/+^ mice (n = 22; *p* < 0.05, *t*-test). (**c**) Synaptic glutamate levels, revealed by γ-DGG (500 μM) inhibition of eEPSC amplitude, are enhanced in P8 *Lgi1*^−/−^ (n = 6) compared to *Lgi1*^+/+^ mice (n = 7; *p* < 0.05, *t*-test).

To further gain insight into the synaptic compartment at play in the Lgi1 modulation of excitatory transmission, we investigated the subcellular localization of Lgi1 with electron microscopy in the hippocampus of wild type mice. To avoid the non-specificity of certain commercial antibodies in immunostaining experiments^15^, we used the CSF containing antibodies against LGI1 from a patient with autoimmune encephalitis and confirmed its specificity on mouse brain (Supplementary Fig. S1). The Lgi1 protein signal was detected in thin neurites, presumably axons, and in some presynaptic terminals, but was never found in postsynaptic structures (Fig. 4a).

We thus investigated whether presynaptic alterations cause the increase in glutamatergic transmission in *Lgi1*^−/−^ mice at P8. A coefficient of variation (CV) analysis of AMPAR eEPSCs revealed an increase in the inverse of the square of the CV (CV^−2^) in *Lgi1*^−/−^ mice at P8 (*Lgi1*^−/−^, n = 23; *Lgi1*^+/+^, n = 22; *p* < 0.05, *t*-test; Fig. 4b). Because such index is correlated with the number of quanta contributing to the evoked EPSC, this suggests a presynaptic origin of the increased transmission in *Lgi1*^−/−^ mice. To further confirm this hypothesis, we directly searched for a change in synaptic glutamate levels in *Lgi1*^−/−^ mice, by using γ-D-glutamylglycine (γ-DGG), a low-affinity competitive antagonist of AMPARs, at a non-saturating concentration (500 μM) at which its potency depends on glutamate levels. γ-DGG inhibition of evoked AMPAR EPSCs was weaker in pyramidal cells from *Lgi1*^−/−^ mice than from *Lgi1*^+/+^ littermates (*Lgi1*^−/−^, n = 6; *Lgi1*^+/+^, n = 7; *p* < 0.05, *t*-test, Fig. 4c), demonstrating that Lgi1-deficiency results in increased synaptic glutamate levels. The increased CV^−2^ and synaptic glutamate levels were not attributable to a higher number of glutamatergic release sites onto *Lgi1*^−/−^ pyramidal cells, as revealed by analysis of excitatory synapse density in hippocampal CA1 area (Fig. 2c,d). They were not due either to an increase in release probability, since paired-pulse ratio of evoked AMPAR EPSCs was also unchanged in pyramidal cells from *Lgi1*^−/−^ mice (*Lgi1*^−/−^: 1.79 ± 0.07, n = 7; *Lgi1*^+/+^: 1.91 ± 0.09, n = 12; *p* > 0.05, *t*-test). These data thus suggest an increased quantal size in hippocampal pyramidal cells from *Lgi1*^−/−^ mice at P8.

Collectively, our results suggest that the increased excitatory synaptic transmission in *Lgi1*^−/−^ mice occurring before seizure onset results from a presynaptic dysfunction leading to an increased release of glutamate at synapses.

### Hippocampal networks are spontaneously hyperexcitable in *Lgi1*^−/−^ mice

**Figure 5:**
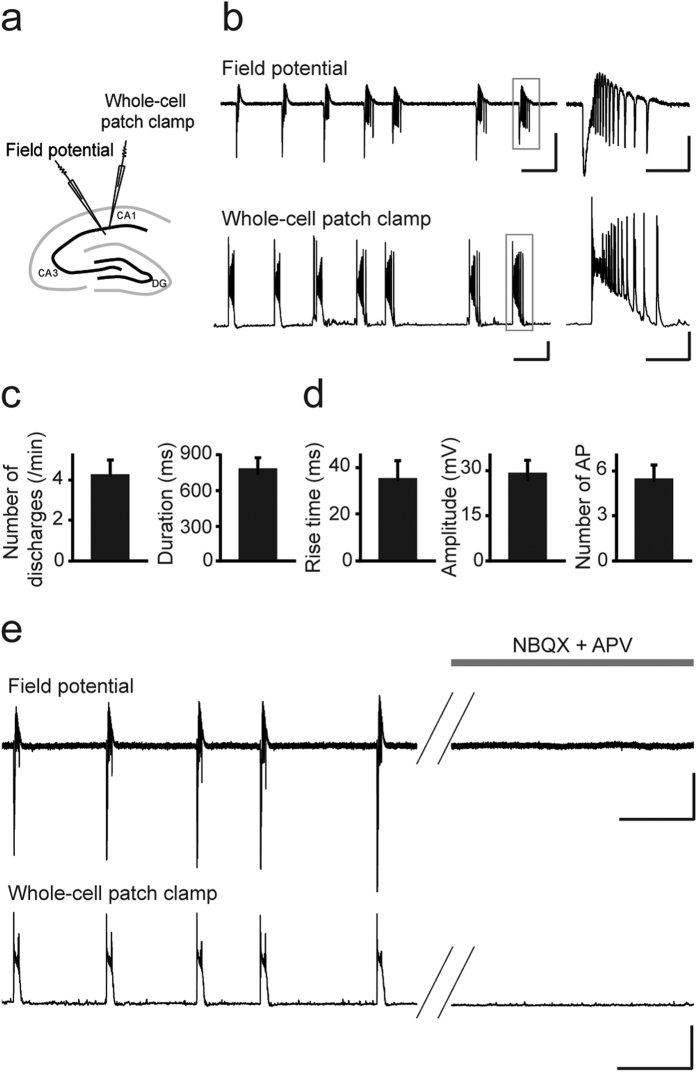
*Lgi1*^−/−^ mice display glutamatergically-driven spontaneous interictal-like discharges. (**a**) Scheme of a hippocampal slice illustrating the arrangement of electrodes for field potential (FP) and whole-cell patch clamp dual recordings. (**b**) Representative trace of CA1 FP (top) and simultaneous whole-cell patch-clamp recording (bottom) of an *Lgi1*^−/−^ CA1 pyramidal cell (resting potential, −70 mV), showing spontaneous interictal-like activities at P9 in normal ACSF. Scale bars: FP: 200 μV, 5 s; whole-cell recording: 20 mV, 5 s. Right: Expanded traces showing an interictal-like network activity (top) that coincides with an intracellular depolarizing plateau potential with burst firing (bottom). Scale bars: FP: 100 μV, 1 s; whole-cell recording: 20 mV, 1 s. Quantification of (**c**) interictal activity and (**d**) depolarizing plateau potential in *Lgi1*^−/−^ CA1 pyramidal cells (n = 14). (**e**) Representative traces of CA1 FP (top) and simultaneous pyramidal cell intracellular recordings (bottom) (resting potential, −70 mV) in an *Lgi1*^−/−^ slice before (left) and after application (right) of glutamate receptor antagonists NBQX (10 μM) and APV (40 μM), which entirely block interictal-like discharges. Scale bars: FP: 100 μV, 10 s; whole-cell recording: 50 mV, 10 s. AP, action potential.

The enhanced synaptic glutamate levels of *Lgi1*^−/−^ mice may impact network activities, leading to a hyperexcitable state preceding seizure onset. To test this hypothesis, we simultaneously performed CA1 extracellular field potential and whole cell current-clamp recordings of CA1 pyramidal cells from P9 *Lgi1*^−/−^ and *Lgi1*^+/+^ littermates (Fig. 5a). Strikingly, in the absence of any pharmacological treatment, we observed, both at network and single pyramidal cell levels, spontaneous interictal-like discharges in hippocampal slices from *Lgi1*^−/−^ mice (Fig. 5b), but not from *Lgi1*^+/+^ mice. Interictal-like network activity was detected in 14 out of 17 slices, occurring at a frequency of ∼4 /min and lasting ∼800 ms (Fig. 5c). Intracellularly, it was characterized by depolarizing plateau potentials with bursts of action potentials (AP) (n = 14; Fig. 5d). Interestingly, these interictal-like discharges in *Lgi1*^−/−^ slices were observed at P9, while ictal activities were never recorded at that stage. Hyperexcitability of hippocampal networks was not due to changes in intrinsic membrane properties of CA1 pyramidal cells, since their membrane capacitance (*Lgi1*^−/−^: 92 ± 6 pF, n = 17; *Lgi1*^+/+^: 91 ± 10 pF, n = 14), input resistance (*Lgi1*^−/−^: 319 ± 31 MΩ, n = 17; *Lgi1*^+/+^: 320 ± 33 MΩ, n = 14), resting membrane potential (*Lgi1*^−/−^: −66 ± 1 mV, n = 14; *Lgi1*^+/+^: −65 ± 1 mV, n = 10), as well as AP amplitude (*Lgi1*^−/−^: 88 ± 2 mV, n = 17; *Lgi1*^+/+^: 91 ± 2 mV, n = 14) and threshold (*Lgi1*^−/−^: −49 ± 1 mV, n = 17; *Lgi1*^+/+^: −48 ± 1 mV, n = 14) were similar in *Lgi1*^−/−^ and *Lgi1*^+/+^ mice (*p* > 0.05, Mann-Whitney). Interestingly, interictal-like activities were fully blocked by antagonists of AMPA (NBQX, 2,3-dihydroxy-6-nitro-7-sulfamoyl-benzo[f]quinoxaline-2,3-dione) and NMDA (APV, (2*R*)-amino-5-phosphonovaleric acid) receptors (Fig. 5e). This indicates that the aberrant network activities specifically recorded in *Lgi1*^−/−^ mice are mediated by glutamatergic synaptic transmission, which is consistent with the increased synaptic glutamate levels in these mice (Fig. 4c). Altogether, our data suggest that the increased glutamatergic drive in Lgi1-deficient mice leads to a hyperexcitable hippocampal network preceding seizure onset.

## Discussion

Inherited focal epilepsies have long been considered as channelopathies. With the involvement of Lgi1, a secreted protein of unknown function, novel mechanisms of epileptogenesis may emerge. We have previously shown that Lgi1-deficient mice are a pertinent model to study spontaneous epileptic activity^12^,^15^. In the present study, we aimed to get insight into the pathogenic mechanisms induced by Lgi1-deficiency. To directly evaluate the very early alterations in neurophysiology caused by Lgi1-deficiency, we assessed hippocampal changes occurring in *Lgi1*^−/−^ mice at early postnatal stages (P8-P9), before first seizures occur and cause damage at cellular and network levels. We combined structural and functional explorations to provide evidence that Lgi1 is a key negative regulator of excitatory synaptic transmission that acts presynaptically. Our data show that Lgi1-deficiency leads early on to increased levels of synaptic glutamate and to hyperexcitability of hippocampal networks, which are thus prone to subsequent ictal activities. This study thus reveals an unexpected likely mechanism of epileptogenesis involved in LGI1-related epilepsies. This mechanism may also be common to ADEAF caused by newly discovered mutations in the *RELN* gene, encoding the reelin, another secreted protein^17^.

Our results suggest that seizure emergence is unlikely to be caused by developmental morphological alterations in hippocampal dendritic and synaptic network. Consistently, no difference was found in CA1 pyramidal cell capacitance, resting membrane potential and input resistance from *Lgi1*^−/−^ mice at the early postnatal stage studied. Supporting this interpretation, autoimmune encephalitis due to LGI1 antibodies appears during adulthood^4^,^5^ and late-postnatal deletion of *Lgi1* induces spontaneous seizures in adult mice^15^, demonstrating that epileptic activities emerge in absence of neurodevelopmental rearrangements. Our finding that Lgi1-deficiency has no major effect on neuronal modeling differs from the one obtained with mouse overexpression of a truncated form of Lgi1, which causes impaired pruning of spines and dendrites, but does not trigger spontaneous seizures^8^,^9^. Probably, *Lgi1* deletion and dominant-negative overexpression induce different cellular mechanisms and have distinct effects.

The involvement of Lgi1 in glutamatergic synaptic transmission has been shown in several studies and models^8^,^13^,^14^,^15^, although it remained unclear whether Lgi1 either facilitates or depresses excitatory transmission. We speculate that the discrepancies between previous studies may be due to the history of seizures in *Lgi1*^−/−^ mice aged P14–P18. Indeed at these stages, during the active phase of epilepsy, seizure-induced cellular and network damage (namely dentate granule cell dispersion, mossy fiber sprouting, neuronal death and astrocytic reactivity^12^) may hamper the detection of defects directly caused early on by *Lgi1* deletion. To clarify the direct effect of Lgi1-deficiency on neuronal activity, independently of alterations induced by ictal activity, we assessed excitatory synaptic transmission in hippocampal slices of mice aged P8–P9, before detection of the first seizures. In this context, we found an enhancement of hippocampal excitatory synaptic transmission resulting from presynaptic, but not postsynaptic, dysfunction. Consistent with these data, and in agreement with Lgi1 being presynaptically secreted^18^, we here showed that Lgi1 is localized in presynaptic terminals and axons, as assessed using LGI1 antibodies contained in CSF from a patient with limbic encephalitis.

Importantly, we showed that synaptic glutamate levels are increased in the hippocampus of *Lgi1*^−/−^ compared to *Lgi1*^+/+^ mice during early postnatal development. We speculate that increased excitatory transmission may be the initial event triggering seizures later on. How Lgi1 acts at presynaptic terminals to regulate glutamate release remains to be elucidated. One hypothesis is the link between Lgi1 and the presynaptic voltage-gated Kv1 potassium channels, since Lgi1 assembles to presynaptic Kv1 channels and inhibits their inactivation 7, and Kv1 channels are major regulators of hippocampal excitability, controlling neurotransmitter release^19^. Adam22, the major receptor of Lgi1, and PSD-95 are not only postsynaptic but also axonal proteins associated with Kv1 channels^20^. Lgi1 may therefore have a dual function through its binding to Adam22 and PSD-95: Kv1-mediated modulation of glutamatergic transmission presynaptically during early postnatal development, and regulation of postsynaptic AMPA receptors trafficking later on^6^,^13^,^18^,^21^. Alternatively, Lgi1 might be related to the synaptic vesicle machinery involved in releasing glutamate, since Lgi1 was shown to interact with several synaptic vesicle proteins such as synaptotagmin, synaptophysin and syntaxin1A^22^. Accordingly, our data suggest an increased quantal size in hippocampal pyramidal cells from *Lgi1*^−/−^ mice.

Remarkably, our data showed that hippocampal slices from P9 *Lgi1*^−/−^ mice displayed spontaneous interictal-like discharges, without any pharmacological treatment. Albeit rarely observed in other animal models of epilepsy, this feature was also reported in *Arx* knock-in (KI) mice^23^. Interestingly, *ARX* is an another epilepsy-related gene encoding a transcription factor which regulates *LGI1* expression^24^. As in *Lgi1*^−/−^ mice, aberrant activities from hyperexcitable network in *Arx* KI mice were shown to result from enhanced glutamatergic drive^23^.

Overall, our study demonstrates how epilepsy genes can deliver critical insights into novel mechanisms underlying epilepsy, which may encompass a spectrum of disorders from inherited temporal lobe epilepsies to severe forms of antibody-mediated encephalitis. While defects in synaptic inhibition have been mostly incriminated in genetic epilepsies^25^, early enhancement of excitatory synaptic transmission may underlie network hyperexcitability in *Lgi1*^−/−^ mice and prone them to seize later on. Increased synaptic glutamate levels resulting early on from presynaptic alterations might thus be an important pathogenic mechanism causing seizures. Our findings may thus open the path to develop new class of antiepileptic drugs targeting synaptic release.

## Methods

### Animals

Heterozygous *Lgi1*^+/−^ mice^12^ on a C57Bl/6 J background were intercrossed to generate *Lgi1*^−/−^, *Lgi1*^+/−^ and *Lgi1*^+/+^ littermates. ICM animal care and use program was approved by the Ministry of Agriculture under the license number A75-1319. All experiments were carried out in strict accordance with the guidelines of the European Directive 2010/63/EU and the French Decree n° 2013-118 concerning the protection of animals used for scientific purposes. All experimental procedures were approved by the ICM Ethics Committee Charles Darwin C2EA-05 and the Ministry of Research under the project number 03539.02. All efforts were made to minimize the number of animals and their suffering. All experiments were performed before seizure onset in P8-P9 age-matched littermates of either sex.

### Electrophysiology

Acute transverse hippocampal slices (300–400 μm) were prepared as previously described^26^,^27^. Slices were transferred to a submerged recording chamber mounted on an Olympus BX51WI microscope and were perfused with an artificial cerebrospinal fluid (ACSF) saturated with 95% O_2_ and 5% CO_2_, containing (in mM): 119 NaCl, 2.5 KCl, 2.5 CaCl_2_, 1.3 MgSO_4_, 1 NaH_2_PO_4_, 26.2 NaHCO_3_ and 11 glucose (pH 7.4). Recordings were acquired using a Multiclamp 700B amplifier (Molecular Devices), digitized at 10–20 kHz, filtered at 2–10 kHz and analyzed using pCLAMP10 and Clampfit softwares (Molecular Devices).

CA1 pyramidal cells were recorded in voltage-clamp mode, using 5–10 MΩ glass pipettes filled with (in mM): 107.5 Cs-gluconate, 20 HEPES, 0.2 EGTA, 8 Na-gluconate, 8 TEA-Cl, 4 Mg-ATP, 0.3 Na3-GTP, and 5 QX314 (pH 7.3; 280–290 mOsm). All the voltage-clamp experiments were performed in the presence of picrotoxin (100 μM). Spontaneous miniature excitatory postsynaptic currents (mEPSCs) were recorded at −70 mV in the presence of 0.5 μM tetrodotoxin (TTX). Evoked excitatory postsynaptic currents (eEPSCs) were induced by stimulating Schaffer collaterals (0.1 Hz) in CA1 *stratum radiatum* with ACSF filled glass pipettes. Evoked AMPAR-mediated EPSCs were measured at −70 mV, while NMDAR-mediated EPSCs were measured at +40 mV when the AMPAR-mediated EPSC had fully decayed. Paired-pulse facilitation of AMPAR EPSCs was induced by delivery of two stimuli at 40 ms interpulse intervals. The average mEPSCs charge transfer was calculated using the Clampfit software. The coefficient of variation (CV) of AMPA eEPSC amplitudes was calculated as: standard deviation/mean.

Intrinsic properties of CA1 pyramidal neurons were measured in current-clamp mode using 4–8 MΩ glass pipettes filled with (in mM): 130 K-methylSO_4_, 5 KCl, 5 NaCl, 10 HEPES, 2.5 Mg-ATP, and 0.3 GTP (pH 7.3; 265–275 mOsm). 2% neurobiotin was added to the pipette solution for neuronal reconstruction. Series resistances were continuously monitored throughout the experiments. Cells were discarded when series resistance varied by more than 20%. Extracellular recordings of field potentials were performed with a glass pipette (∼1 MΩ) filled with ACSF and positioned in CA1 *stratum radiatum*, ∼100 μm away from the recorded pyramidal cell.

### Neuronal reconstruction

Hippocampal slices with neurobiotin-filled neurons were fixed in 4% paraformaldehyde phosphate buffer saline, then blocked for endogenous peroxidases (3% H_2_O_2_ and 10% methanol) and permeabilized in 2% Triton X-100. Slices were then incubated in avidin-biotinylated horseradish peroxidase in 1% Triton X-100 followed by diaminobenzidine (DAB) peroxydase and finally transferred to slides, dehydrated in ethanol and mounted with Eukitt. Detailed shape of neurobiotin-filled CA1 pyramidal cells was imaged using a CX 9000 Olympus microscope and analyzed using the Neurolucida software (MicroBrightField).

### Western blot

Hippocampi were dissected and lysed as previously reported^15^. 20 μg of proteins was separated on 10% Bis-Tris polyacrylamide gels (NuPAGE, Invitrogen) and transferred to nitrocellulose (iBlot, Invitrogen). Western Blot analysis was performed using rabbit polyclonal anti-GluR1 (1 μg/ml, ab31232, Abcam), rabbit polyclonal anti-GluR2/3 (1/1000, AB1506, Millipore) and anti-α-actin (1/1000, A2066, Sigma-Aldrich) primary antibodies. Quantification was done using Multi Gauge densitometry software.

### Electron microscopy

P8 mice were anesthetized with an intraperitoneal injection of a lethal dose of sodium pentobarbital and transcardiacally perfused with 2% paraformaldehyde/1% glutaraldehyde in 0.1 M phosphate buffer (PB), pH 7.4. Brains were removed and postfixed in 4% paraformaldehyde. Coronal slices (45 μm) were cut using a vibratome. Briefly, hippocampi were microdissected and postfixed in 1% osmium tetroxide, incubated in 1% uranyl acetate, dehydrated in ethanol, and embedded in Epon. Ultrathin sections (50 nm) were cut, contrasted with uranyl acetate and lead citrate, and analyzed with an electron microscope (JEOL, 1200 EX II). Density of visually identified asymmetrical synapses, length of postsynaptic density (PSD) and number of docked vesicles (within 30 nm from the synaptic membrane of the active zone) were measured using ImageJ software on acquired images (30.000 × magnification) from the thick medial part of CA1 *stratum radiatum*. The surface area evaluated per animal was 2900 μm^2^.

### Immunohistochemistry

P8 or P15 mice were anesthetized with an intraperitoneal injection of a lethal dose of sodium pentobarbital and perfused with 4% paraformaldehyde in 0.1 M PB, pH 7.4. Brains were removed and post-fixed in the same fixative for 24 hours at 4 °C. Cryostat coronal slices of 16 μm thickness were immersed in citrate buffer 0.01 M for heat induced epitope retrieval. Standard procedures of immunohistochemistry were performed. Primary antibodies used were rabbit polyclonal anti-GluR1 (AB1504, Millipore) and mouse monoclonal anti-extracellular GluR2 (MAB397, Millipore) antibodies. An undiluted CSF from a patient with limbic encephalitis containing LGI1 antibodies (as assessed by clinical diagnosis) was used on brain sections from *Lgi1*^+/+^ and *Lgi1*^−/−^ littermate mice (aged P15). DAB peroxidase (HRP) substrate kit (Vector laboratories) was used to reveal staining.

### Immunostaining for electron microscopy

Hippocampi were incubated before post-fixation in 1% osmium tetroxide in an undiluted CSF (same than above) from a patient with limbic encephalitis containing antibodies against LGI1. Hippocampi were then incubated in biotinylated goat anti-human secondary antibody (BA-3000, Vector laboratories), in avidin-biotinylated horseradish peroxidase and in DAB peroxidase.

### Statistics

All data are expressed as mean ± SEM. Statistical significance for between-group comparisons was determined by unpaired Student’s *t*-test or nonparametric Mann-Whitney test. Statistical significance for within-group comparisons was determined by two-way ANOVA. The Kolmogorov-Smirnov test was used for cumulative distribution comparison. Statistical analysis was performed in GraphPad Prism and Statistica.

## Additional Information

**How to cite this article**: Boillot, M. *et al.* LGI1 acts presynaptically to regulate excitatory synaptic transmission during early postnatal development. *Sci. Rep.* **6**, 21769; doi: 10.1038/srep21769 (2016).

## Supplementary Material

Supplementary Fig. S1
