# Supplementary material for: LGI1 acts presynaptically to regulate excitatory synaptic transmission during early postnatal development
Source: Sci Rep. 2016 Feb 16;6:21769. doi: 10.1038/srep21769 (PMC4754946; doi:10.1038/srep21769)
Supplement: Supplementary Fig. S1 [file srep21769-s1.pdf]

LGI1 acts presynaptically to regulate excitatory synaptic transmission during early postnatal development

Morgane Boillot[¤], Chun-Yao Lee[¤], Camille Allene, Eric Leguern, Stéphanie Baulac[§*] and Nathalie Rouach[§*].

[¤] MB and CYL equally contributed to the work. [§*] SB and NR are co-last authors and corresponding authors.

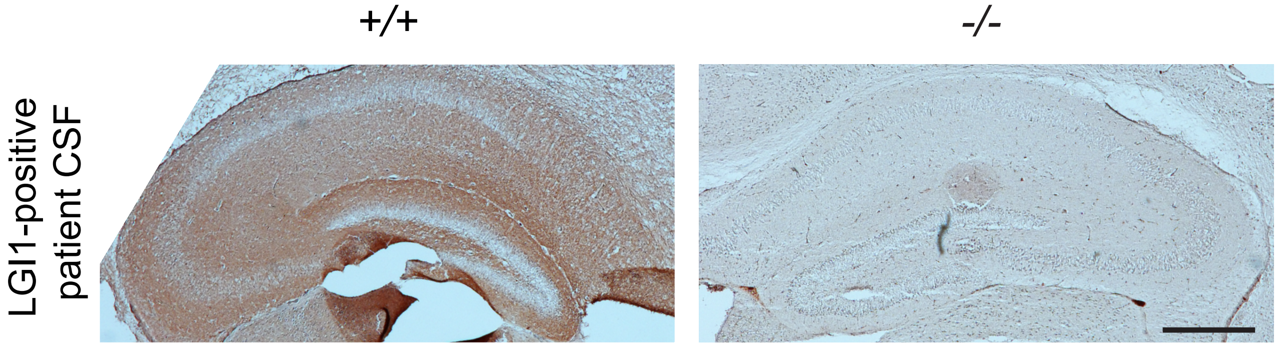

Supplementary Figure S1. LGI1 antibodies associated with limbic encephalitis specifically label Lgi1 in mice.

Immunostaining with CSF from LGI1-positive patient on hippocampal coronal sections from *Lgi1+/+* (n = 2) and *Lgi1-/-* (n = 2) mice. Scale bar: 400 µm.
